# Altered reactive oxygen species scavenging and hormonal signaling in tetraploid rice are associated with blast resistance

**DOI:** 10.1093/plphys/kiae547

**Published:** 2024-10-16

**Authors:** Ningning Wang, Chenxi Wang, Keyan Liu, Zitian Leng, Yingkai Wang, Weilong Meng, Dayong Li, Chunying Zhang, Jian Ma

**Affiliations:** Faculty of Agronomy, Jilin Agricultural University, Changchun 130117, China; Faculty of Agronomy, Jilin Agricultural University, Changchun 130117, China; Faculty of Agronomy, Jilin Agricultural University, Changchun 130117, China; Jilin Provincial Laboratory of Crop Germplasm Resources, Jilin Agricultural University, Changchun 130117, China; Faculty of Agronomy, Jilin Agricultural University, Changchun 130117, China; Faculty of Agronomy, Jilin Agricultural University, Changchun 130117, China; Jilin Provincial Laboratory of Crop Germplasm Resources, Jilin Agricultural University, Changchun 130117, China; Faculty of Agronomy, Jilin Agricultural University, Changchun 130117, China; College of Plant Protection, Jilin Agricultural University, Changchun 130117, China; Faculty of Agronomy, Jilin Agricultural University, Changchun 130117, China; Faculty of Agronomy, Jilin Agricultural University, Changchun 130117, China; Jilin Provincial Laboratory of Crop Germplasm Resources, Jilin Agricultural University, Changchun 130117, China

## Abstract

Autotetraploid rice shows distinct morphological, physiological, hormonal, and gene expression changes that enhance its resistance to rice blast.

Dear Editor,

Rice blast, caused by *Magnaporthe oryzae*, substantially undermines global food security by drastically reducing rice (*Oryza sativa* L.) yields (Cui et al. 2021). Traditional mitigation strategies, encompassing selective breeding and chemical interventions, are increasingly compromised by the pathogen's rapid adaptation, diminishing both the efficacy of fungicides and the durability of resistance in rice varieties (Borrelli et al. 2018; Jiang et al. 2019). Polyploidization, stemming from whole genome duplication, has emerged as a promising strategy to augment resistance in polyploid rice against *M. oryzae* (Bundo and Coca 2016).

The enhanced resilience of polyploid rice is attributed to genetic modulations of reactive oxygen species (ROS) production and phytohormone signaling pathways; however, the underlying mechanisms orchestrating these changes remain largely enigmatic. Our study seeks to bridge this gap through in-depth metabolomic and transcriptomic analyses, uncovering distinctive adaptive mechanisms employed by autotetraploid rice against *M. oryzae* infection. Through comparative assessments of diploid (GFD-2X) and its autotetraploid counterpart (GFD-4X), our findings demonstrate that GFD-4X exhibits superior tolerance, characterized by markedly milder symptoms and reduced infection rates, while maintaining biomass post-infection (Fig. 1, A to G).

**Figure 1.**
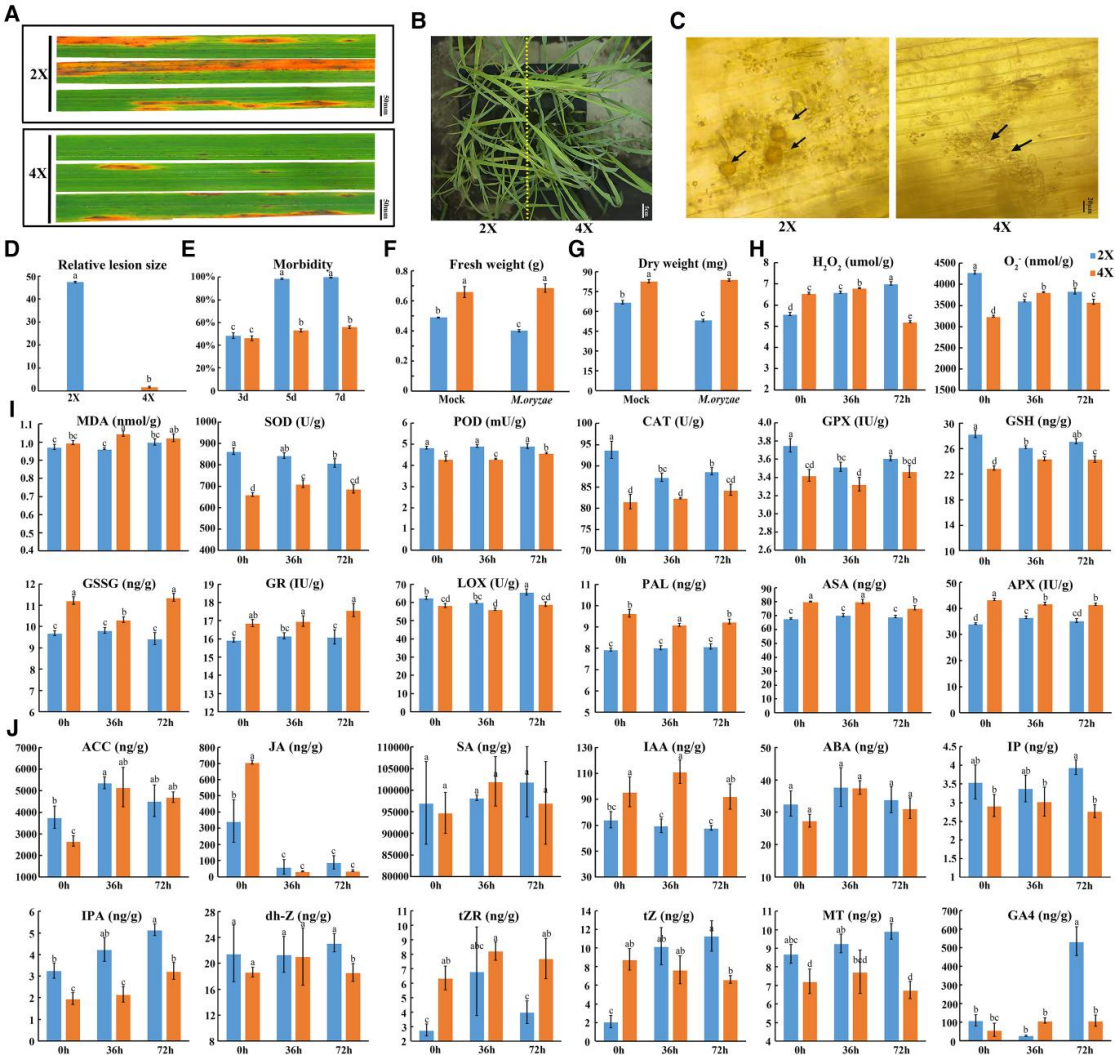
Morphological and physiological responses of GFD-2X and GFD-4X to *Magnaporthe oryzae* infection. This bar chart illustrates the comprehensive changes in phytohormones and physiological indices over infection time for 2 rice genotypes, GFD-2X and GFD-4X, in response to *M. oryzae* infection. **A)** Phenotypic changes in 2-wk-old GFD-2X and GFD-4X seedlings, sprayed with a *M. oryzae* conidial suspension (1 × 10^5^ conidia/mL in 0.2% Tween-20), captured 7 d post-infection (dpi). **B)** Infection status in GFD-2X and GFD-4X captured 5 dpi. **C)** Spore germination of *M. oryzae* in GFD-2X and GFD-4X at 36 h post-infection. **D)** The extent of infection damage. **E)** Morbidity rates at 3, 5, and 7 dpi. **F)** Fresh and **G)** dry weights measured at 5 dpi. **H)** Changes in physiological indices pre- and post-infection, including hydrogen peroxide (H_2_O_2_), superoxide (O_2_^−^). **I)** MDA, malondialdehyde; GSSG, glutathione oxidized and enzymatic activities (SOD, superoxide dismutase; POD, peroxidase; CAT, catalase; GPX, glutathione peroxidase; GR, glutathione reductase; GSH, glutathione reductase; LOX, lipoxygenase; PAL, L-phenylalanine ammonia-lyase; ASA, ascorbic acid; APX, ascorbate peroxidase) measured at 0, 36, and 72 h post-inoculation (hpi). **J)** Changes in phytohormones or their precursors, including 1-aminocyclopropane-1-carboxylic acid (ACC, the direct precursor to ethylene), jasmonic acid (JA), salicylic acid (SA), indole-3-acetic acid (IAA), abscisic acid (ABA); N6-isopentenyladenine (IP), isopentenyl adenosine (IPA, a cytokinin precursor or derivative), dihydrozeatin (dh-Z), trans-Zeatin-riboside (tZR), trans-Zeatin (tZ), melatonin (MT), and gibberellin A_4_ (GA_4_), observed at 0, 36, 72 hpi. 2X and 4X indicate trends for GFD-2X and GFD-4X, respectively. Each data set is derived from 3 biological replicates, the different letters above the columns represent significant differences (*P* < 0.05) on the basis of Duncan’s new multiple range method.

Biochemical assessments revealed that in GFD-4X, hydrogen peroxide (H_2_O_2_) levels were significantly heightened at both 0 and 36 h post-inoculation (hpi) compared to GFD-2X, suggesting a robust priming response, akin to the findings of Wang et al. (2021) regarding enhanced salt tolerance in tetraploid rice. Notably, a sharp decline in H_2_O_2_ levels at 72 hpi (Fig. 1H) highlights the presence of an adept ROS scavenging system within the tetraploid rice, potentially crucial for curtailing oxidative damage over prolonged stress exposure (Mittler et al. 2022). This dynamic in ROS management is further corroborated by substantial elevations in oxidative stress markers such as malondialdehyde (MDA), glutathione disulfide (GSSG), glutathione reductase (GR), L-phenylalanine ammonia-lyase (PAL), ascorbic acid (ASA), and ascorbate peroxidase (APX) in GFD-4X relative to its diploid counterpart (Fig. 1I). Effective scavenging of H_2_O_2_ through elevated activities of ROS scavenging enzymes in autotetraploid rice not only helps mitigate its detrimental effects on photosynthesis but also contributes to an equilibrium between pathogen defense and robust plant growth. The variations in these physiological and hormonal indicators between GFD-2X and GFD-4X are depicted in Supplementary Fig. S1. The enhanced growth and development observed in GFD-4X, evidenced by its significantly higher biomass compared to GFD-2X (Fig. 1, F and G), may be attributable to its bolstered ROS scavenging efficacy.

Comparative phytohormone profiling revealed nuanced shifts in the hormonal landscape of GFD-4X compared to GFD-2X, marked by substantial decreases in levels of jasmonic acid (JA), N6-isopentenyladenine (IP), isopentenyl adenosine (IPA), dihydrozeatin (dh-Z), melatonine (MT), and gibberellin A_4_ (GA_4_). Conversely, there were notable increases in indole-3-acetic acid (IAA) and trans-zeatin-riboside (tZR) (Fig. 1J). These alterations corroborate prior findings on the functional role of these phytohormones in rice defense against *M. oryzae* (Helliwell et al. 2016; Qiu et al. 2022) and the specific impact of IAA on enhancing blast resistance (Domingo et al. 2009). These observations illuminate the sophisticated physiological and hormonal adaptation strategies GFD-4X employs to fortify its defense architecture.

Transcriptional profiling of GFD-2X and GFD-4X in response to *M. oryzae* infection yielded 131.62 Gb of clean data, facilitating robust bioinformatics analyses. By 36 and 72 hpi, a substantial shift in gene expression was observed from the baseline (0 hpi), with GFD-4X displaying considerably more differentially expressed genes (DEGs) (Fig. 2A; Supplementary Table S1). This trend aligns with the enhanced blast resistance observed in the allotetraploid rice (Fig. 2B; Supplementary Table S2). This was further evidenced by the analysis of unique and co-expressed DEGs, illustrating complex genetic adaptations at varying infection stages and ploidy levels (Fig. 2, C and D; Supplementary Table S2). Validation using reverse transcription quantitative PCR (RT-qPCR) confirmed a strong correlation (R^2^ = 0.8606) between RNA-seq data and RT-qPCR results, substantiating the reliability of our transcriptional findings (Supplementary Fig. S2; Supplementary Table S3).

**Figure 2.**
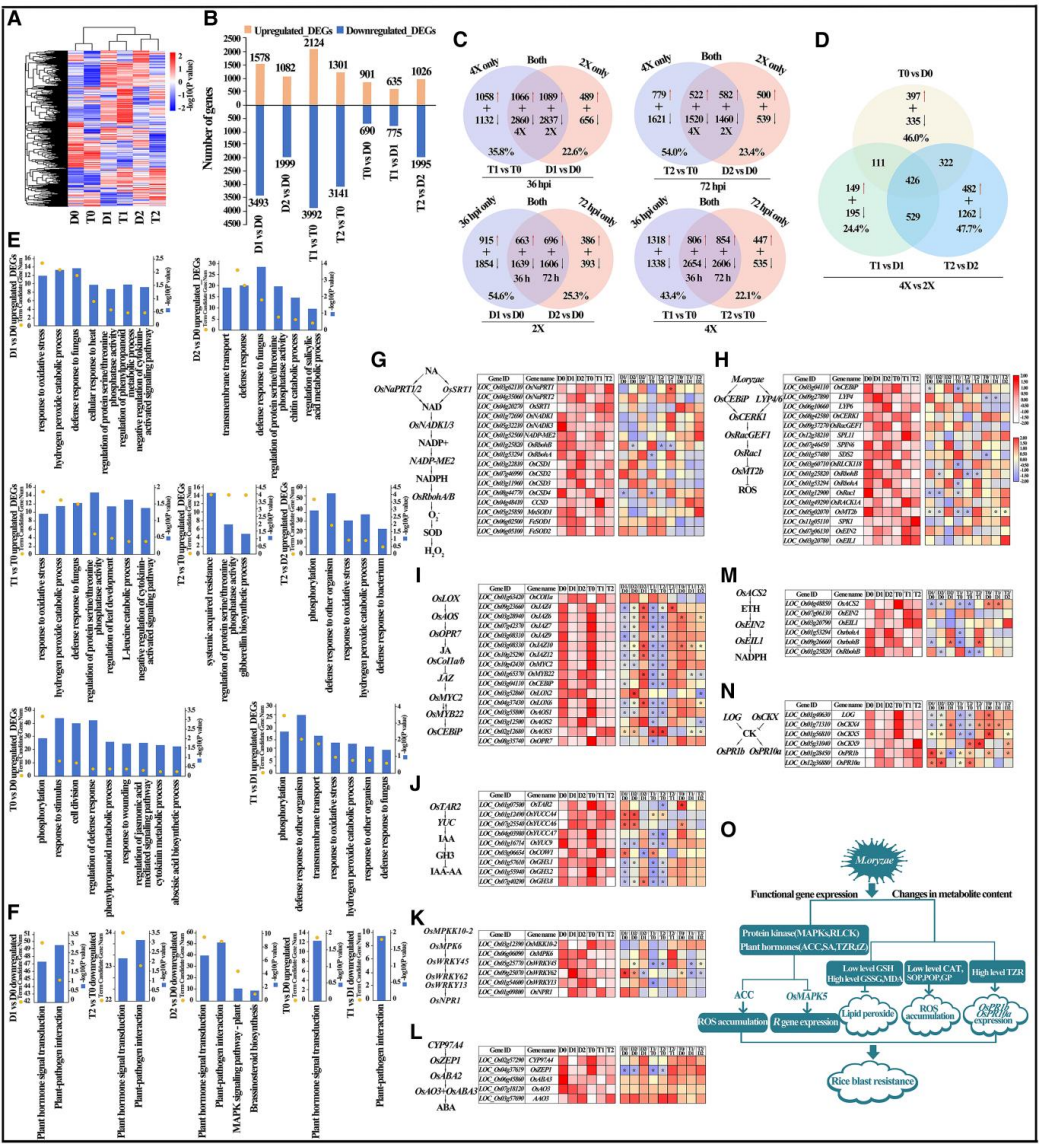
Temporal gene expression patterns in autotetraploid and diploid rice following *Magnaporthe oryzae* infection. This figure provides a detailed analysis of gene expression in autotetraploid rice (GFD-4X) and its diploid counterpart (GFD-2X) in response to *M. oryzae* infection. **A)** Heat map displaying hierarchical clustering of overall gene expression in leaves. **B)** Charts of differentially expressed genes (DEGs) comparing responses in GFD-2x and GFD-4x leaves to *M. oryzae* infection, highlighting the number of upregulated (orange lines) and downregulated (blue lines) DEGs. **C and D)** Venn diagrams illustrating both shared and unique gene expressions in GFD-2X and GFD-4X. The red and black arrows represent upregulated and downregulated DEGs, respectively. **E)** GO enrichment analyses of upregulated DEGs post-infection in GFD-2X and GFD-4X. **F)** KEGG enrichment analyses of DEGs post-infection in GFD-2X and GFD-4X. The *y* axis represents the negative log10-transformed *P* value (blue lines) and gene numbers (yellow dots). **G and H)** Transcriptomic analysis of genes within the Reactive Oxygen Species (ROS)-regulated network in rice under pathogen attack, featuring schematic diagrams of ROS biosynthesis and the defense regulation, gene expression profiles related to ROS synthesis. **I to N)** Examination of gene expression in the phytohormone-regulated network post-infection, including schematic diagrams of phytohormone biosynthesis pathways, gene expression profiles related to phytohormone synthesis, and correlations between phytohormone content and gene expression. The white color represents the expression levels of genes (in log2 transformed read counts per million), while the yellow color indicates the fold change (log2 value) of the DEGs. Statistical significance is denoted by asterisks (*) with *P* < 0.05, based on Student's *t*-test. **O)** Schematic representation of signal transduction mechanisms in autotetraploid rice in response to *M. oryzae* infection, delineating pathways with stimulatory (arrows) and inhibitory (“T” shapes) interactions. D0, D1, and D2 represent 0, 36, and 72 h post-inoculation (hpi) in diploid rice, respectively, while T0, T1, and T2 represent 0, 36, and 72 hpi in autotetraploid rice, respectively.

Gene Ontology (GO) and Kyoto Encyclopedia of Genes and Genomes (KEGG) pathway analyses revealed substantial variations in pathway enrichment crucial for blast resistance, differing by infection time and ploidy (Fig. 2E; Supplementary Fig. S3). Upregulated genes were involved in pathways like phosphorylation, H_2_O_2_ catabolic process, and defense responses, while downregulated genes focused on stress responses (Supplementary Table S4). Notably, pathways regulating protein serine/threonine phosphatase activities increased post-infection, whereas the MAPK cascade pathway was downregulated (Fig. 2, E and F; Supplementary Tables S4 and S5).

Upon inoculation, GFD-4X upregulated the key component of the NAD salvage pathway essential for ROS synthesis, while downregulating the ROS scavengers (Fig. 2, G and H; Supplementary Table S6). These differential expressions corroborate our biochemical findings of more rapid accumulations of ROS and MDA accumulation in GFD-4X (Fig. 1I), indicative of a more robust ROS-mediated defense mechanism. Enzymes critical for regulating ROS homeostasis, such as NADPH oxidase and superoxide dismutase (Torres 2010; Gupta et al. 2021), were also markedly upregulated in GFD-4X compared to its diploid counterpart (Fig. 2, G and H; Supplementary Table S6), highlighting the strategic importance of this defense pathway in enhancing blast resistance.

Phytohormone signaling pathways, including those for JA, ET, and CK, also showed notable changes (Fig. 2, I to N; Supplementary Table S7), aligning with their recognized roles in signaling perception and mediation of defense systems in response to *M. oryzae* infection (De Vleesschauwer et al. 2013). Specifically, cytokinin oxidase (CKX) genes, which are responsible for CK degradation, were more pronouncedly downregulated in GFD-4X, resulting in elevated CK accumulation and enhanced defense signaling. This was associated with the upregulation of numerous pathogen-related genes in GFD-4X, such as pathogenesis-related protein 1b (*OsPR1b*) and pathogenesis-related protein 10a (*OsPR10a)* (Fig. 2N), further validating the enhanced defensive capability.

Moreover, GO and KEGG analyses highlighted a marked enhancement in the protein phosphorylation pathway in GFD-4X (Supplementary Fig. S4, Supplementary Table S8), particularly within the MAPK pathway, as evidenced by marked enrichment in comparison groups D2 vs. D0, T1 vs. T0, and T2 vs. T0 (Supplementary Fig. S5 and Supplementary Table S9). This aligns with the rapid activation of the MAPK signaling cascade, triggering a vigorous defense response through ROS production and gene activation (Ding et al. 2022). This highlights the adaptive strategies of polyploid rice in response to *M. oryzae* infection, emphasizing coordinated signal transduction and gene expression in plant immunity (Fig. 2O).

In conclusion, our results suggest that tetraploid rice exhibits a more dynamic and early engagement of signaling pathways, particularly the MAPK cascade, upon pathogen invasion, which may contribute to its enhanced priming response and blast resistance. While this study highlights the potential of polyploidization to strengthen plant resistance against pathogens in rice, suggesting it could aid crop improvement and sustainable agriculture, it is important to note that our findings might be genotype-specific. Therefore, these results should be interpreted with caution until more generalized impacts can be confirmed.

## Supplementary Material

kiae547_Supplementary_Data

## Data Availability

The data sets generated and analyzed in this study are available at PRJNA1026748 (https://www.ncbi.nlm.nih.gov/bioproject/PRJNA1026748 [accessed on October 11, 2023]).

## Supplementary data

The following materials are available in the online version of this article.

**Supplementary Figure S1.** Correlation matrices of physiological and biochemical parameters in rice plants under different ploidy levels.

**Supplementary Figure S2.** Validation of RNA-seq results by RT-qPCR confirms differential gene expression between autotetraploid rice (GFD-4X) and its diploid counterpart (GFD-2X) in response to *M. oryzae* infection.

**Supplementary Figure S3.** GO enrichment analyses of downregulated DEGs post-infection in GFD-2X and GFD-4X.

**Supplementary Figure S4.** Transcriptomic analysis of genes involved in protein phosphorylation signaling pathways in GFD-2X and GFD-4X following *M. oryzae* infection.

**Supplementary Figure S5.** Transcriptomic analysis of genes involved in MAPK signaling pathways in GFD-2X and GFD-4X following *M. oryzae* infection.

**Supplementary Table S1.** RNA-seq data of GFD-2X diploid and tetraploid rice under *M. oryzae* infection.

**Supplementary Table S2.** The transcriptome analysis results compare the differentially expressed genes (DEGs) in GFD-2X and GFD-4X following *M. oryzae* infection.

**Supplementary Table S3.** Primers for RT-qPCR used in this study.

**Supplementary Table S4.** GO analysis results of differentially expressed genes (DEGs) in GFD-2X and GFD-4X following *M. oryzae* infection.

**Supplementary Table S5.** KEGG analysis results of differentially expressed genes (DEGs) in GFD-2X and GFD-4X following *M. oryzae* infection.

**Supplementary Table S6.** Gene expression profiles involved in the ROS regulatory pathway in GFD-2X and GFD-4X following *M. oryzae* infection.

**Supplementary Table S7.** Gene expression profiles involved in the plant hormones pathway in GFD-2X and GFD-4X following *M. oryzae* infection.

**Supplementary Table S8.** Gene expression profiles involved in the Protein phosphorylation pathway in GFD-2X and GFD-4X following *M. oryzae* infection.

**Supplementary Table S9.** Gene expression profiles involved in the MAPK signaling pathway in GFD-2X and GFD-4X following *M. oryzae* infection.

## Dive Curated Terms

The following phenotypic, genotypic, and functional terms are of significance to the work described in this paper:

NAD CHEBI: CHEBI:13389

NADPH CHEBI: CHEBI:16474

MDA Gramene: Malondialdehyde
MDA Araport: Malondialdehyde
