# Supplementary material for: Altered reactive oxygen species scavenging and hormonal signaling in tetraploid rice are associated with blast resistance
Source: Plant Physiol. 2024 Oct 16;197(1):kiae547. doi: 10.1093/plphys/kiae547 (PMC11663496; doi:10.1093/plphys/kiae547)
Supplement: kiae547_Supplementary_Data [file kiae547_supplementary_data.zip › Supplementary Data.pdf]

SUPPLEMENTARY DATA

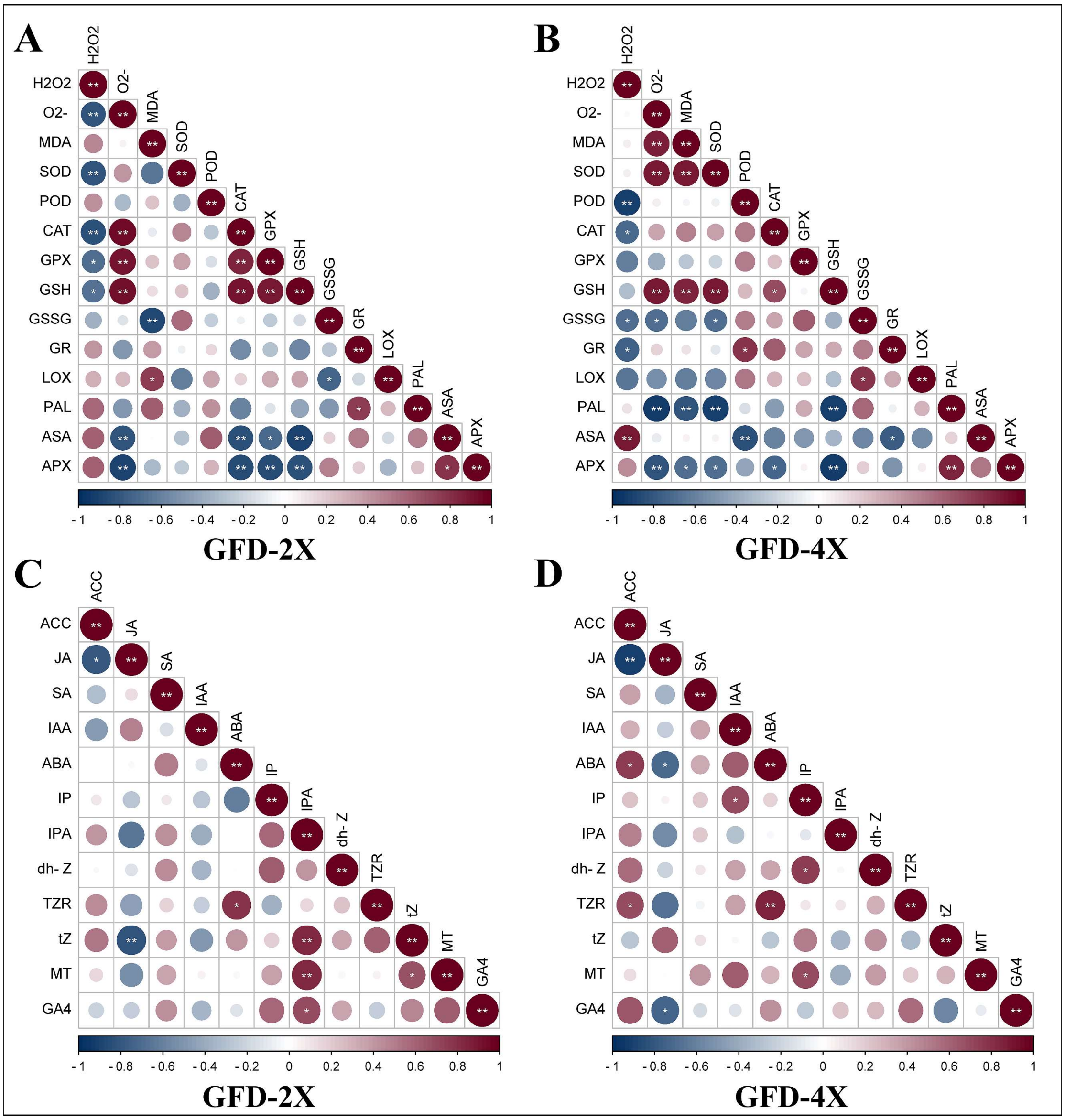

Supplementary Figure S1. Correlation matrices of physiological and biochemical parameters in rice plants under different ploidy levels. The matrices display Pearson correlation coefficients between the parameters. Positive correlations are shown in red, and negative correlations in blue, with the intensity and size of the circles indicating the strength of the correlation. Significant correlations are marked with asterisks, *$p < 0.05$, ** $p < 0.01$ respectively, based on Student's t-test. **(A)** Correlation matrix for physiological and biochemical parameters in diploid rice (GFD-2X), focusing on oxidative stress markers (H_2O_2, O_2^-, MDA), antioxidant enzymes (SOD, POD, CAT, GPX, APX), non-enzymatic antioxidants (GSH, GSSG, ASA), and other enzymes (GR, LOX, PAL). **(B)**

Correlation matrix for the same parameters as in (A) in allotetraploid rice (GFD-4X). **(C)** Correlation matrix for hormonal parameters in diploid rice (GFD-2X), including ethylene precursor (ACC), jasmonic acid (JA), salicylic acid (SA), indole-3-acetic acid (IAA), abscisic acid (ABA), isopentenyladenine (IP), isopentenyladenosine (IPA), dihydrozeatin (dh-Z), trans-zeatin riboside (tZR), trans-zeatin (tZ), melatonin (MT), and gibberellic acid (GA4). **(D)** Correlation matrix for the same hormonal parameters as in (C) in allotetraploid rice (GFD-4X). The ploidy levels GFD-2X and GFD-4X represent diploid and allotetraploid rice, respectively. The analysis helps understand the complex interactions between physiological, biochemical, and hormonal responses under different ploidy conditions.

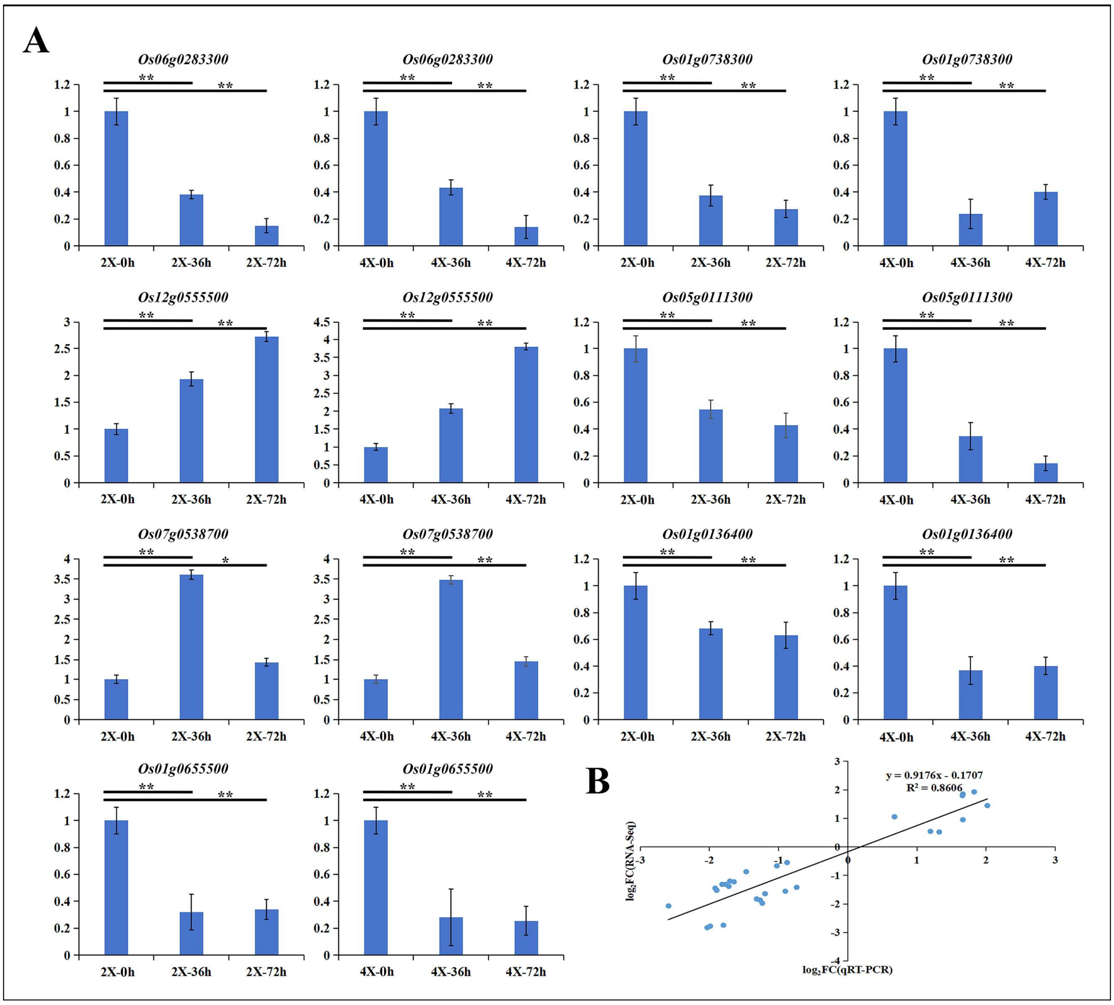

Supplementary Figure S2. Validation of RNA-seq results by RT-qPCR confirms differential gene expression between autotetraploid rice (GFD-4X) and its diploid counterpart (GFD-2X) in response to *M. oryzae* infection. (A) The results of seven genes expression amplified by RT-qPCR. The y-axis represents expression level and x-axis represents the samples of diploid and autotetraploid rice after rice blast infection. The type of error line is the standard deviation (SD). * and ** indicate the level of significant differences between treatment and control groups at $p < 0.05$ and $p < 0.01$, respectively, based on Student's t-test. **(B)** Comparison of RNA-seq results and RT-qPCR analysis of gene expression levels.

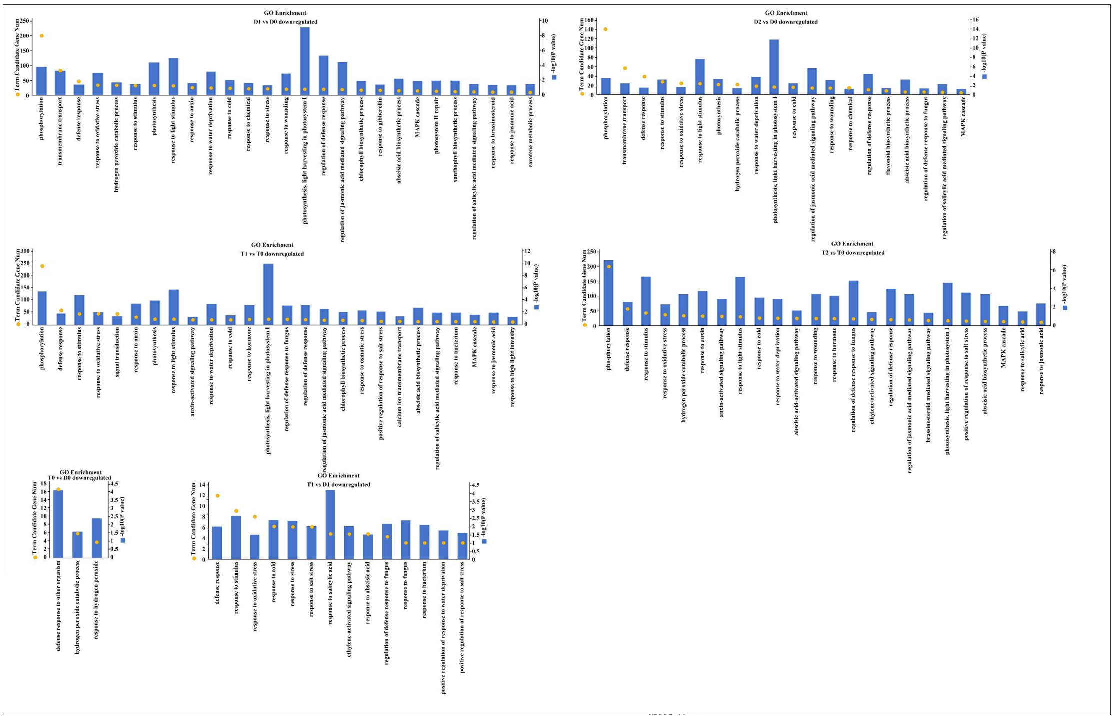

Supplementary Figure S3. GO enrichment analyses of downregulated DEGs post-infection in GFD-2X and GFD-4X. The y-axis represents the negative log10-transformed P value (blue lines) and gene numbers (yellow dots). D0, D1, and D2 represent 0, 36, and 72 hours post-inoculation (hpi) in diploid rice, respectively, while T0, T1, and T2 represent 0, 36, and 72 hpi in autotetraploid rice, respectively.

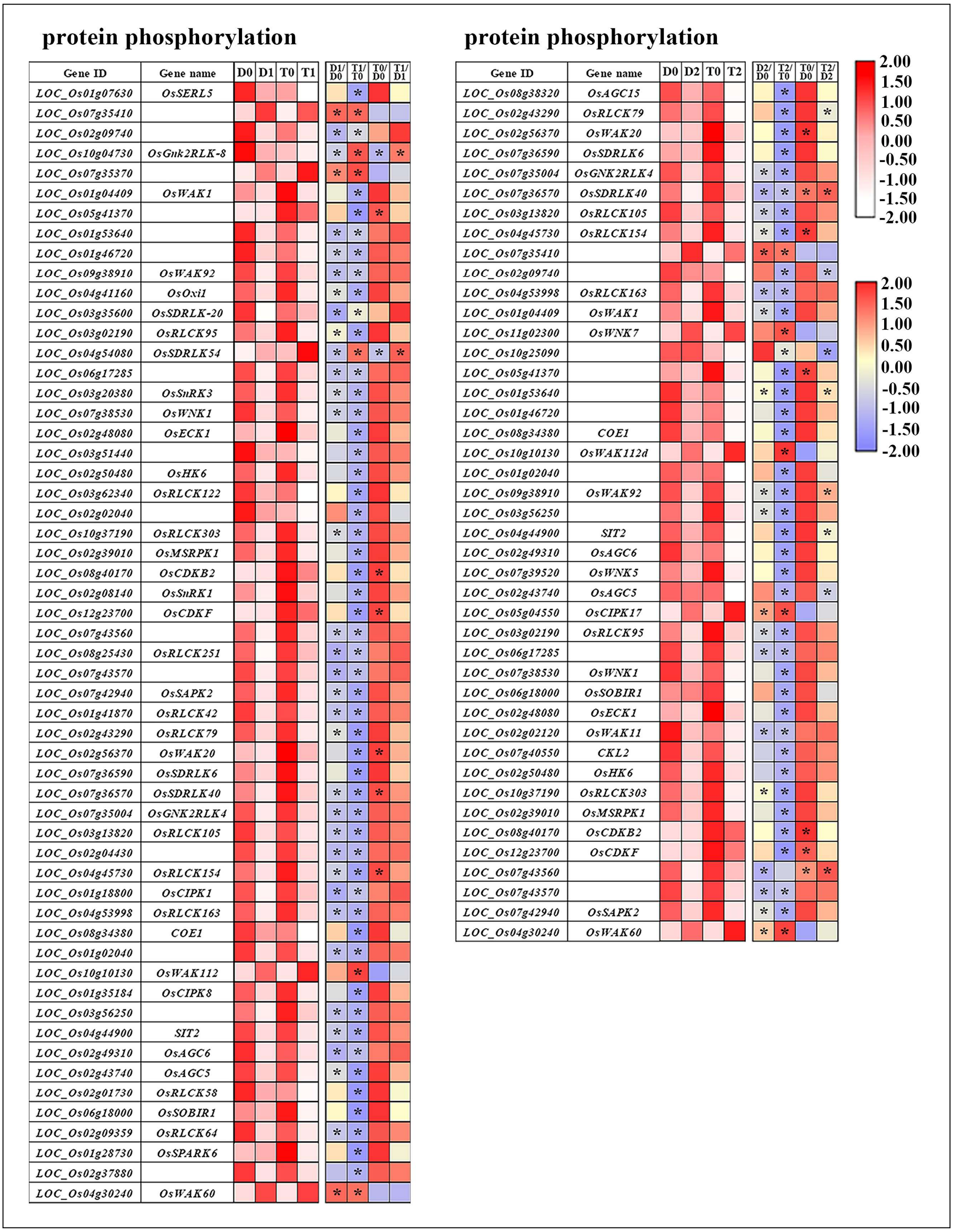

Supplementary Figure S4. Transcriptomic analysis of genes involved in protein phosphorylation signaling pathways in GFD-2X and GFD-4X following *M. oryzae* infection. The white color represents the expression levels of genes (in log2 transformed read counts per million), while the yellow color indicates the fold change (log2 value) of the DEGs. Statistical significance is denoted by asterisks (*) with $p < 0.05$, based on Student's t-test. D0, D1, and D2 represent 0,

36, and 72 hours post-inoculation (hpi) in diploid rice, respectively, while T0, T1, and T2 represent 0, 36, and 72 hpi in autotetraploid rice, respectively.

MAPK signaling pathway

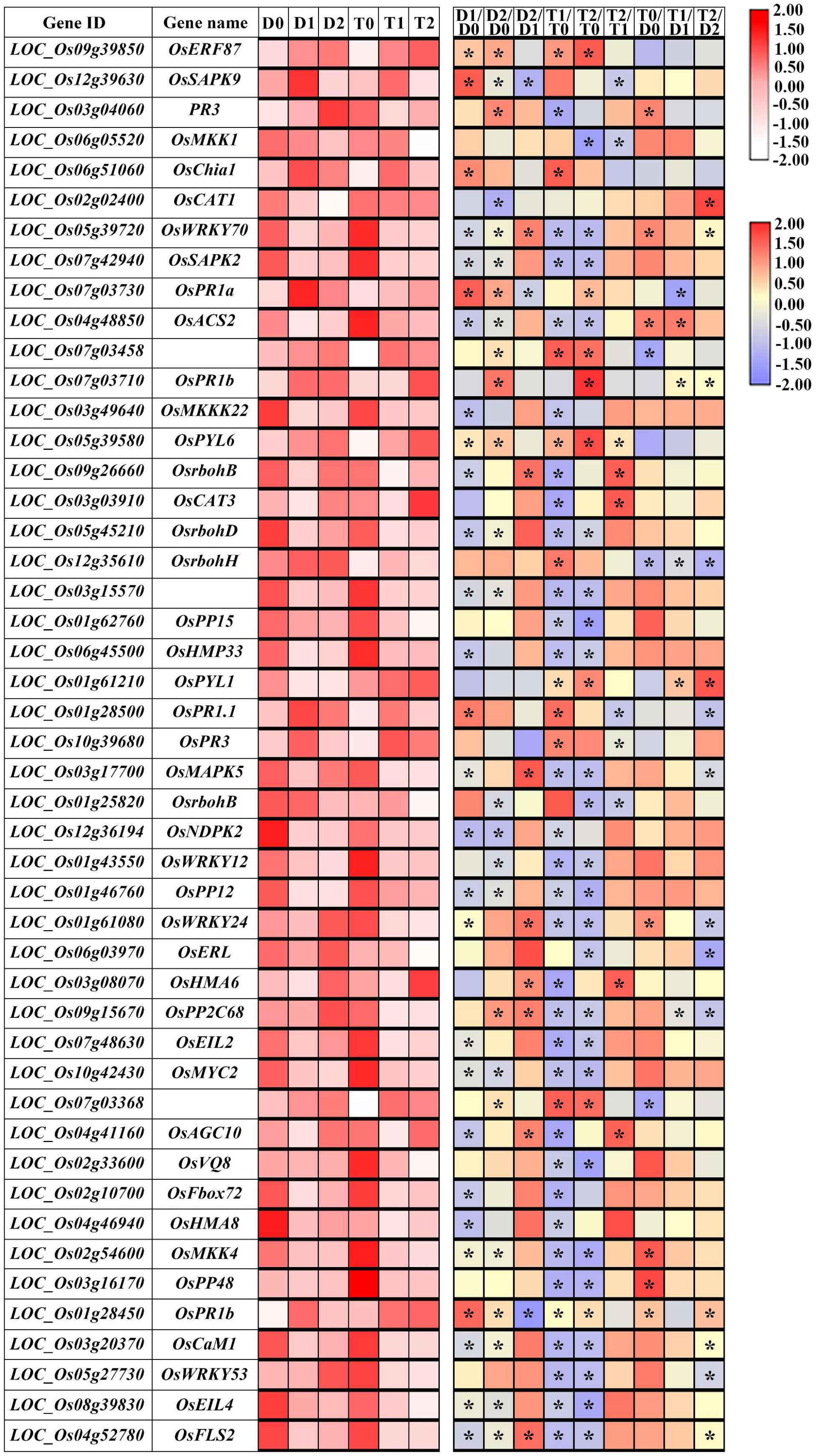

Supplementary Figure S5. Transcriptomic analysis of genes involved in MAPK signaling pathways in GFD-2X and GFD-4X following *M. oryzae* infection. The white color represents the expression levels of genes (in log2 transformed read counts per million), while the yellow color indicates the fold change (log2 value) of the DEGs. Statistical significance is denoted by asterisks (*) with $p < 0.05$, based on Student's t-test. D0, D1, and D2 represent 0, 36, and 72 hours post-inoculation (hpi) in diploid rice, respectively, while T0, T1, and T2 represent 0, 36, and 72 hpi in autotetraploid rice, respectively.
